# Effects of short-term repeated sprint training in hypoxia or with blood flow restriction on response to exercise

**DOI:** 10.1186/s40101-022-00304-1

**Published:** 2022-09-03

**Authors:** Margaux Giovanna, Robert Solsona, Anthony M. J. Sanchez, Fabio Borrani

**Affiliations:** 1 Institute of Sport Sciences, University of Lausanne, Lausanne, Switzerland; 2 Faculty of Sports Sciences, Laboratoire Interdisciplinaire Performance Santé Environnement de Montagne (LIPSEM), University of Perpignan Via Domitia, UR 4640, 7 Avenue Pierre de Coubertin, 66120 Font-Romeu, France

**Keywords:** Hypoxic training, Exercise training, Maximal oxygen consumption, Skeletal muscle, Vascular occlusion

## Abstract

This study compared the effects of a brief repeated sprint training (RST) intervention performed with bilateral blood flow restriction (BFR) conditions in normoxia or conducted at high levels of hypoxia on response to exercise. Thirty-nine endurance-trained athletes completed six repeated sprints cycling sessions spread over 2 weeks consisting of four sets of five sprints (10-s maximal sprints with 20-s active recovery). Athletes were assigned to one of the four groups and subjected to a bilateral partial blood flow restriction (45% of arterial occlusion pressure) of the lower limbs during exercise (BFRG), during the recovery (BFRrG), exercised in a hypoxic room simulating hypoxia at FiO_2_ ≈ 13% (HG) or were not subjected to additional stress (CG). Peak aerobic power during an incremental test, exercise duration, maximal accumulated oxygen deficit and accumulated oxygen uptake (*VO*_2_) during a supramaximal constant-intensity test were improved thanks to RST (*p* < 0.05). No significant differences were observed between the groups (*p* > 0.05). No further effect was found on other variables including time-trial performance and parameters of the force-velocity relationship (*p* > 0.05). Thus, peak aerobic power, exercise duration, maximal accumulated oxygen deficit, and *VO*_2_ were improved during a supramaximal constant-intensity exercise after six RST sessions. However, combined hypoxic stress or partial BFR did not further increase peak aerobic power.

## Introduction

In the last two decades, high-intensity interval training (HIT) has gained remarkable popularity in the sports sphere. Similar or higher gains in several variables, including maximal oxygen uptake ($\dot{V}{O}_2\mathit{\max}$), muscle oxidative capacity and time trial performance were shown compared to endurance training protocols achieved with higher training volume [1–3]. The addition of hypoxic stimulus to HIT has been increasingly acknowledged to favor training adaptations [4–9]. Performing exercise bouts under systemic hypoxia reduces arterial oxygen partial pressure and thus increases energy production through substrate-level phosphorylation [10] and adaptations such as enhanced mitochondrial density, oxidative enzyme activity, and capillary density [11–13]. However, it is well recognized that the use of systemic hypoxia induces several adaptive responses and may improve both aerobic and anaerobic performance, as well as repeated sprint ability depending on the nature of the training protocol administered [14, 15]. In the last decade, it was found that repeated sprint training (RST) in hypoxic conditions (called “RSH”) may promote several additional effects on adaptations to exercise [16]. Thus, improvements in repeated sprint ability and several sport-specific physical and technical parameters have been recently reported, notably in swimmers and tennis players [17, 18].

Importantly, it was observed that RSH can significantly enhance performance in a very short window period. In the study from Beard et al., the authors found that four sessions of RSH spread over 2 weeks enhanced repeated sprint ability (i.e., both maximal and mean power) in world-level male rugby union players [19]. Other studies also highlighted that intensified training over 2 weeks or RSH over 10 days can improve physical performance in soccer players and a professional cyclist, respectively [20, 21]. Finally, another investigation demonstrated that six sessions of RSH spread over 2 weeks improved repeated sprint ability of upper body muscles in cross-country skiers [22]. Thus, these findings make RSH of practical relevance in several sports disciplines, especially those where only a short window period is available prior to competition (i.e., “in-season”).

On the other hand, some studies found no impact of RSH on field performance in swimmers [23] or endurance performance in rugby players [24]. Another point of concern is the loss of exercise performance observed in some HIT acute studies in hypoxia, which could lead to relative undertraining compared to training in normoxia in chronic studies [25, 26]. However, those studies used longer sprint exercises than RST. Thus, as glycogenolysis rates decline during long sprints, performance could be more impaired in these protocols [27]. Altogether, RSH potentially improves training adaptations during training protocols.

In addition to training under systemic hypoxia, other strategies have been proposed to enhance training adaptations with local hypoxia in muscles such as training with blood flow restriction (BFR) [28, 29]. BFR training is becoming increasingly popular and represents another way to generate local hypoxia in skeletal muscles and/or to limit venous return depending on occlusion pressure. In recent years, the impact of BFR on performance has been increasingly studied and several investigations have suggested that using BFR may be suitable to provide additional physiological stress and to improve performance.

A study showed that a short-term low-intensity interval BFR (140 to 200 mmHg) training simultaneously increases maximum oxygen uptake ($\dot{V}{O}_2\mathit{\max}\Big)$ and muscle strength, but no effect was found with HIT even if combined with BFR [30]. Importantly, BFR is enjoying a resurgence in popularity especially since it was combined with HIT protocols [31–33]. Thus, Mitchell et al. [34] recently highlighted that adding partial BFR to a 4-week sprint interval training program enhanced $\dot{V}{O}_2\mathit{\max}$. In this study, HIT without BFR did not show these benefits [34]. Nonetheless, effects on critical power were the same with or without BFR and no impact was found on skeletal muscle mitochondrial and capillarity protein content in either group [34]. Of note, Taylor and coworkers have previously reported that sprint interval training with post-exercise BFR was an effective stimulus to augment $\dot{V}{O}_2\mathit{\max}$ but the authors did not find improvement in exercise performance (i.e. 15-km time trial) [33]. Furthermore, little is known about the use of BFR during RST protocols except for acute physiological responses. It was suggested that the intermittent application of BFR during RST promotes greater changes in skeletal muscle blood volume (as observed by an increase of total hemoglobin concentrations) [31]. Given this fact, RST with BFR could be considered as an alternative to RSH to induce similar or complementary adaptations and potentially enhance performance.

Hence, even if progress has been made on the impact of training with BFR on performance, there is a lack of data regarding the effects of RST with BFR. Even though continuous BFR has already been compared to intermittent BFR [35], to the best of our knowledge, no study has tested the effects of BFR during exercise versus recovery. Indeed, it has been shown that intermittent BFR can induce similar responses and adaptations compared to continuous BFR, with a reduced pain perception [36, 37]. In addition, no study has compared the benefits of training with BFR to those of training in systemic hypoxia in a single study, especially during a short window period. Nonetheless, achieving performance gains quickly is of practical relevance in several sport disciplines where only a brief period is available prior to championship [19]. Therefore, the present study aimed to compare the effects of RST with bilateral partial BFR (45% of femoral artery occlusion pressure measured at rest) with a systemic hypoxic training protocol at 13% FiO_2_ (4000 m simulated altitude) on both aerobic and anaerobic responses to exercise (i.e., ramp incremental exercise, time trial, torque-velocity test, and supramaximal exercise) in a short window period. It has been chosen to set maximal stress for both hypoxic and BFR protocols that could be tolerated in this cohort in combination with the training sessions proposed based on preliminary work in our laboratory. Moreover, this study also compared two BFR protocols (i.e., BFR during exercise versus BFR during recovery). It was expected that BFR during exercise would induce higher local stress among muscles and would be more beneficial than BFR during recovery.

## Materials and methods

### Participants

A priori power analyses were performed with alpha and beta errors of 0.05 and 0.20, respectively, and a medium effect size (*f* = 0.25) as it has been observed during these training protocols [4–9]. The required sample size was 36 split into four groups. Hence, forty male endurance-trained participants initially volunteered to be enrolled in the study and 39 were included in the analysis (mean ± SD; age 25.6 ± 5.7 years; height 176.0 ± 4.4 cm; weight 79.1 ± 15.3 kg; body mass index 23.8 ± 2.5 kg m^−2^) (Table 1). Participants were involved in endurance activities, including running and/or cycling. The participants were not acclimated to altitude and did not sojourn for more than 2 days at altitude during the 2 months preceding the experiment. Participants were instructed to maintain regular dietary habits throughout the period of the protocol and to abstain from alcohol and dietary supplements or ergogenic aids during the studied period. The study procedures complied with the last Declaration of Helsinki on human experimentation and were approved by the “Commission cantonale d’éthique de la recherche sur l’être humain, Canton de Vaud, CER-VD” (number 2016-02068). The participants provided written informed consent and were informed about possible inconveniences associated with the experiment. All experiments were performed in accordance with relevant guidelines and regulations.

**Table 1:** Anthropometric characteristics of the participants

	HG	BFRrG	BFRG	CG	***P value***
**Age (year)**	24.5 ± 4.0	23.5 ± 3.6	23.9 ± 3.8	30.2 ± 9.9	*p = 0.34*
**Height (cm)**	180.4 ± 3.2	178.2 ± 6.1	181.5 ± 3.1	180.7 ± 6.0	*p = 0.53*
**Weight (kg)**	80.6 ± 11.6	73.8 ± 7.1	77.4 ± 8.1	77.4 ± 5.0	*p = 0.44*
**Body mass index (kg**^**.**^**m**^**−2**^**)**	24.8 ± 3.8	23.3 ± 2.2	23.5 ± 2.4	23.7 ± 1.3	*p = 0.76*

Data are shown as mean ± standard deviation. *HG* (*N* = 10) training in hypoxia, *BFRG* (*N* = 10) training with blood flow restriction during exercise, *BFRrG* (*N* = 10) training with blood flow restriction during recovery, CG (*N* = 9), control group with normal training. Analysis of variance (ANOVA) was used to compare differences of means among the four groups

### Study design

**Fig. 1:**
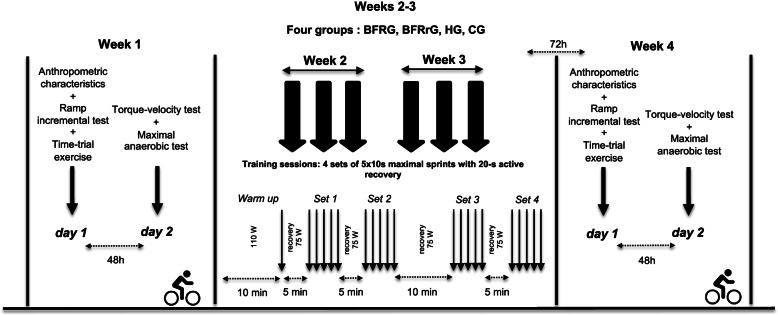
Sequence of the experimental protocol. Participants were randomized into four groups (HG, training in hypoxia; BFRG, training with blood flow restriction during exercise; BFRrG, training with blood flow restriction during recovery; CG, control group without hypoxia or BFR) for a training period of 2 weeks. During warm-up, participants were instructed to exercise at 110 W for 10 min and to finish with a 10-s maximal sprint. On two occasions, participants were asked to perform a battery of tests on two consecutive days

The study was conducted over 4 weeks (Fig. 1) including 1 week of pre-tests (W1), 2 weeks of training (W2 and W3) counting six training sessions (three times per week), and 1 week of post-tests (W4). On W1 and W4, the participants performed a battery of tests in normoxia over 2 days separated by 48 h, including a ramp incremental exercise (day 1), a time trial performance (day 1), a torque-velocity relationship test (day 2), and an anaerobic capacity exercise (day 2). All testing was carried out at the laboratory with the same environmental condition at the same hour of the day to minimize the effects of circadian cycles. The post-tests on W4 were assessed 3 days after the last training session. In addition, all participants were instructed not to perform high-intensity exercises in their personal training to limit interference with the present protocol.

Randomization across the groups was done using $\dot{V}{O}_2\mathit{\max}$ values relative to body weight according to stratified simple randomization method, and the participants were assigned to one of the four RST groups: HG (hypoxic training group; *N* = 10, simulated altitude of 4000 m; FiO_2_ ≈ 13%); BFRG (training with blood flow restriction during sprint exercises in normoxia; *N* = 10), BFRrG (training with blood flow restriction during recovery between sprint exercises in normoxia; *N* = 10), and CG in normoxia (control group; training in normoxic group, *N* = 9).

Randomization was done by an independent researcher blinded to the individuals. BFRG and BFRrG training were subjected to bilateral-leg partial occlusion (45% of femoral artery occlusion pressure measured at rest; 88.2 ± 10.1 mmHg). Of note, the cuff pressure of 45% was fixed following preliminary work performed in the laboratory that highlighted it was the highest level that could be tolerated by the participants in combination with the present RSH protocol. BFR was set up during the 10 s of exercises using automated cuffs. The cuff was released between intervals for this group. For BFRrG, participants were asked to maintain a stationary position on the ergocycle and BFR was applied immediately at the end of each sprint exercise during the 20 s recovery periods. Verbal encouragement was provided during the tests, as well as during the training sessions.

### Tests

#### Anthropometric and femoral artery occlusion pressure measurement

On arrival at the laboratory for the first visit, anthropometric characteristics were assessed using bioelectrical impedance analysis (Tanita, MC-780 MAP, France), and femoral artery occlusion pressure of the lower limbs was measured. For this purpose, automated cuffs (11 × 85 cm cuff size, 10 × 41 cm bladder size, SC10D Rapid Version Cuff, D. E. Hokanson, Inc., Bellevue, WA, USA) were placed on the most proximal region of the lower limbs [38]. Artery pressure was measured at seated rest on a chair by gradually increasing the occlusion pressure until a point when no blood flow was observed in the femoral artery using a linear Doppler ultrasound probe (L12-5L60N) with Echo Wave II 3.4.4 software (Telemed Medical Systems, Lithuania, Telemed Ltd., Milan, Italy). These measurements were performed two or three times to make sure there was no variation and for accuracy, with 2 min between trials. The highest-pressure value obtained was used to determine the 45% of femoral artery occlusion pressure applied for BFRG and BFRrG during the exercise sessions.

#### Ramp incremental test

Participants performed a ramp test on an electronically-braked cycling ergometer (Lode Excalibur Sport Ergometer, Lode B.V., Netherlands). First, participants completed a warm-up which was composed of 3 steps of 4 min each at 75, 105, and 135 W respectively. Then, after 5 min of rest, the ramp incremental test was initiated at 60 W with an increase of 1 W every 2 s, (30 W/min) until exhaustion. Cardio-respiratory variables were measured breath-by-breath using a metabolic cart (Quark, Cosmed, Italy). Breathing flow was measured by a bi-directional digital turbine that was calibrated at the beginning of each test with a 3-L syringe. O_2_ and CO_2_ analyzers were calibrated before each test using ambient air and standard gas (O_2_: 15%, CO_2_: 4.99%). The breath-by-breath pulmonary gas exchange data were collected continuously throughout the test. $\dot{V}{O}_2\mathit{\max}$ and maximum heart rate (*HRmax*) were determined averaging the last 30 s of exercise. HR was collected with a telemetry-based HR monitor (Garmin, Garmin Ltd., Lathe, Kansas, USA). The end of the ramp test was based on the achievement of three of the following criteria [39]: (i) heart rate greater than 90% of the predicted maximum (220 beats per minute−age) (ii) respiratory exchange ratio (RER) ≥ 1.10 [40], (iii) the athlete’s rating of perceived exertion (i.e., Borg Scale rating ≥ 18), and (iv) the participants stopped the exercise despite strong verbal encouragements. At the end of this test, participants performed an active recovery at a self-adjusted low-intensity pace during 10 min on the ergocycle. Power at the end of the test was considered as the peak aerobic power [41]. The height and configuration of the saddle and handlebars were recorded and reproduced in subsequent tests.

#### Time trial

To simulate a field cycling performance, participants performed a 250-kJ time trial on the same ergocycle [42]. Participants were instructed to complete the exercise as fast as possible. The resistance and pedal cadence were self-selected in a linear mode load where the linear factor (*α*) was calculated as follows: *α* = *P*/*rpm*^2^; where *P* (W) is 70% of peak aerobic power and revolutions per minute (rpm) is the theoretical optimal cadence (85 rpm). The work progression in kilojoules was given to the participants.

#### Torque–velocity test

On day 2, participants performed a warm-up consisting of 6 min of submaximal cycling at 100 W, followed by two short submaximal accelerations (5 s) at 80 and 100 rpm, respectively. After 4 min of passive recovery, participants performed a force-velocity test [43] consisting of six repetitive maximal sprints of 5 s in isokinetic mode (i.e., 70, 85, 100, 115, 130, and 145 rpm). The sprints were separated by 4 min of active recovery at 50 W and 80 rpm. Participants had 10 s before the sprint to reach the requested cycling cadence. Torque, rpm, and power were recorded at the frequency of 5 Hz. The torque-velocity relationship was described using a linear relationship. An iterative process was used to minimize the sum of the squared errors between the fitted function and the observed values. Force when contraction velocity is null (*T*_0_, *x*-axis intercept), velocity of unloaded shortening (*v*_0_, *y*-axis intercept), and the slope of the relationship were estimated. The power-velocity relationship was modeled using a second-degree polynomial function where the power of each sprint was the average of the last 3 s of sprinting. In the same way, an iterative process was used to determine the parameter of the quadratic function. The derivative of the polynomial (derivative = zero) was used to calculate the maximum power from this test (*P*_max_) and to deduce the optimal pedaling velocity (*v*_opt_) and torque (*T*_opt_) at *P*_max_.

#### Assessment of anaerobic capacity

The warm-up consisted of three exercises at different powers (60, 90, 120 W) each lasting 4 min, followed by 5 min of passive recovery. Then, participants performed a supramaximal exercise (105% peak aerobic power) to exhaustion on ergocycle acting in hyperbolic (rpm-independent) mode [44–46]. Cardio-respiratory variables were measured breath-by-breath. Anaerobic energy release (i.e., anaerobic capacity) was assessed by quantification of the maximal accumulated oxygen deficit. This consists of calculating the difference over time between the theoretical oxygen consumption at a supra maximum intensity (105% of peak aerobic power) and the effective oxygen uptake. The theoretical consumption was obtained by extrapolating the curve of oxygen consumption as a function of sub-maximum loads. Oxygen consumption of sub-maximum loads was acquired during the warm-up of the ramp incremental test (75 W, 105 W, and 135 W) and during the anaerobic capacity test (60 W, 90 W, and 120 W). Maximal accumulated oxygen deficit was calculated as the area between theoretical and actual oxygen consumption as a function of time. Exercise duration, peak oxygen uptake ($\dot{V}{O}_2 peak$), total O_2_ demand, accumulated oxygen uptake (O_2_ uptake), the contribution of both aerobic and anaerobic processes (%), % $\dot{V}{O}_2\mathit{\max}$, and mean $\dot{V}{O}_2$, were measured. Minute ventilation ($\dot{V}E$), $\dot{V}E$/$\dot{V}\mathrm{O}$_2_, $\dot{V}E$*/*$\dot{V}\mathrm{CO}$_2_*,* RER, and heart rate (HR) at the end of the test are also provided. To minimize artifact, values have been filtered (i.e., second order zero-lag Butterworth filter, cutoff frequency 0.05).

### Training protocol

Participants followed a 2-week training program with three training sessions *per* week [22] according to the modality drawn at random. After warming up for 10 min at 110 W, participants performed a first 10-s maximal sprint followed by an active recovery of 5 min at 75 W. Then, the repeated-sprint protocol consisted of four sets of 5 × 10-s maximal (i.e., all-out and sitting) cycling sprints separated by 20 s of active recovery at 75 W between sprints. Five minutes of active recovery (75 W) were performed between sets 1 and 2, and 3 and 4. Recovery between sets 2 and 3 consisted of 10 min of active recovery at 75 W. Sprints were conducted on an ergocycle programmed on constant torque mode (Wingate mode) with a torque factor of 0.8 Nm.kg^–1^. Shoe clips were used to ensure pedal contact. Exercise bouts were initiated from a rolling start. No progressive overload was applied to training or hypoxic stimulus because the window period was very short, and participants were endurance trained. Mean power, maximal power, and total work were collected throughout the protocol. In addition, a fatigue index was adapted from previous study [47] for each sprint:

$$Fatigue\ index=100\ast \frac{Total\ sprint\ mean\ power}{Ideal\ sprint\ mean\ power}-100$$

where Total sprint mean power corresponds to the sum of sprint mean powers from all sprints and Ideal sprint mean power is the number of sprints x mean power of the most powerful sprint.

Participants were encouraged energetically to complete every repetition maximally and verbal indication of time was provided during each sprint for pacing prevention. Water intake was permitted ad libitum during the exercise sessions.

### Statistics

Data from training sessions are presented as mean values ± standard deviation (SD). Data from the tests are presented graphically as box and whisker plots, including the minimum and maximum values, the interquartile region, the median, and the mean. Quartiles are also shown. Statistical analyses were performed using Jamovi (Version 1.1.9.0). One-way ANOVA (analysis of variance) was used to compare anthropometric characteristics before the training program. Linear mixed models were used for all the other variables. Linear mixed models consider both the nested and crossed structure of the data [48, 49]. Thus, Linear mixed models provide precise estimates when data are hierarchized. After inspecting residual plots, no obvious deviations from normality and homoscedasticity were observed. The fixed effect was the group (HG, BFRG, BFRrG, CG), and the time (pre- and post-tests) where participant was the random effect. To obtain contrasts, Holm correction was used. Significance was declared when *p* < 0.05. Effect sizes (*d*) are also provided (trivial effect *d* < 0.10, small effect *d* = 0.20, medium effect *d* = 0.50 and large effect *d* = 0.8) [50].

## Results

### Training sessions

**Fig. 2:**
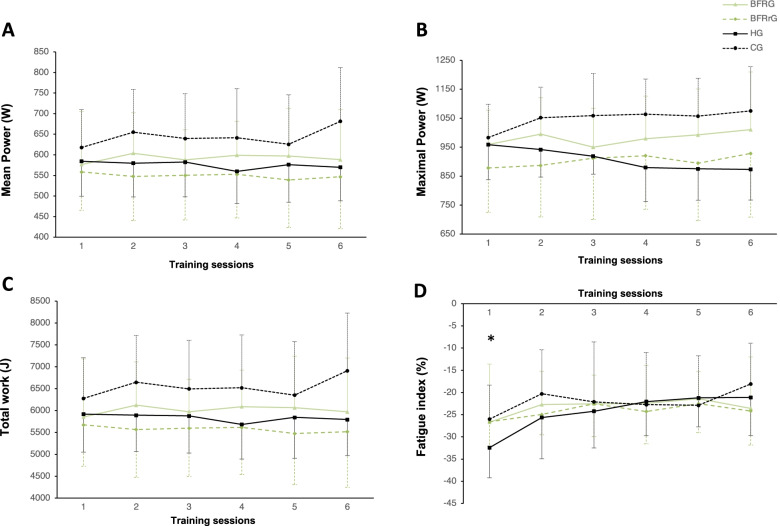
Evolution of mean power (**A**), maximal power (**B**), total work (**C**) and fatigue index (**D**) during the training protocol. HG, training in hypoxia (*N* = 10); BFRG, training with blood flow restriction during exercise (*N* = 10); BFRrG, training with blood flow restriction during recovery (*N* = 10); CG, training in normal group (*N* = 9). *: significantly different from the other training sessions (*p* < 0.05). Of note, for more clarity, standard deviations are displayed unilaterally

There were initially 10 participants per group but one of them stopped the experimentation within CG for personal reasons. There were no symptoms of dizziness during all conditions. There was no significant difference on training data (i.e., mean power, maximal power, and total work; Fig. 2) between groups (*p* = 0.26, 0.09, and 0.26, respectively) and between the different training sessions (*p* = 0.47, 0.29 and 0.50, respectively). An effect of training session was found for fatigue index (*p* < 0.001). Post hoc analyses showed that fatigue index was greater during the first session compared to the second (*p* = 0.011; *d* = 0.58), third (*p* < 0.001; *d* = 0.63), fourth (*p* = 0.001; *d* = 0.62), fifth (*p* < 0.001; *d* = 0.73), and sixth (*p* < 0.001; *d* = 0.71). There was no significant interaction for mean power, total work, and fatigue index (*p* = 0.39, 0.20 and 0.50, respectively). However, an interaction was found for maximal power (*p* = 0.004) even if post hoc analyses did not reveal significant effects after Holm correction.

### Ramp incremental test

**Fig. 3:**
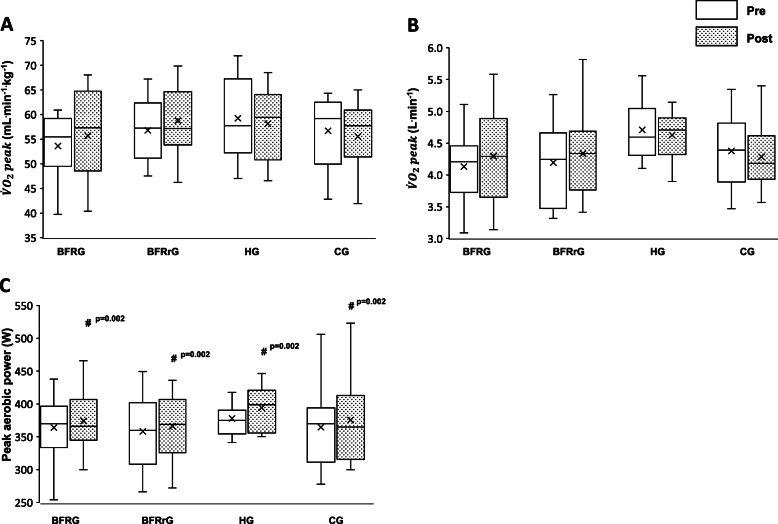
Results of the ramp test. **A** Relative and **B** absolute peak oxygen consumption $(\dot{V}{O}_2 peak)$, and **C** peak aerobic power before and after training in hypoxia (HG, *N* = 10), training with blood flow restriction during exercise (BFRG, *N* = 10), training with blood flow restriction during recovery (BFRrG, *N* = 10), and training in normal group (CG, *N* = 9). # training effect. The whiskers represent minimum and maximum values, the boxes the interquartile region, the horizontal lines the medians, the crosses are the mean. Quartiles are also shown

There was no time and group effect, or interaction, on $\dot{V}{O}_2 peak$ relative to body weight during the ramp exercise (*p* = 0.80, 0.09 and 0.28, respectively). No time and group effect, or interaction, was found for absolute $\dot{V}{O}_2 peak$ values (*p* = 0.52, 0.25, and 0.14, respectively). Peak aerobic power increased in all groups after training (*d* = 0.5, 0.2, 0.2, 0.2 for HG, BFRG, BFRrG, and CG, respectively). This represents an increase with training of 4.4%, 2.8%, 2.2%, and 3.0% for HG, BFRG, BFRrG, and CG, respectively (Fig. 3). Peak aerobic power expressed relative to body weight also increased with time (*p* = 0.008; *d* = 0.3, for HG, and 0.2 for BFRG, BFRrG, and CG). However, no interaction and no group differences were found (*p* = 0.82 and 0.86, respectively) (Fig. 3).

### Time trial performance

**Fig. 4:**
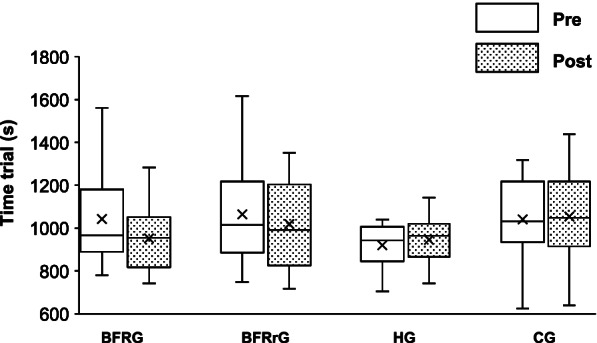
Time trial performance. A Time to reach 250 kJ before and after training in hypoxia (HG, *N* = 10), training with blood flow restriction during exercise (BFRG, *N* = 10), training with blood flow restriction during recovery (BFRrG, *N* = 10), and training in normal group (CG, *N* = 9). The whiskers represent minimum and maximum values, the boxes the interquartile region, the horizontal lines the medians, the crosses are the mean. Quartiles are also shown

At baseline, time to complete the 250 kJ time trial was not significantly different between the groups (*p* = 0.75) and remained unchanged (*p* = 0.14) when repeated after the training program (Fig. 4). There was a significant interaction (*p* = 0.042) for mean power, however, the adjusted *p* value (Holm correction) was not significant (*p* = 0.19).

### Torque-velocity exercise

No significant effect of training was found concerning *P*_max_, *T*_0,_ v_0_, *T*_opt_, and *v*_opt_ (Table 2). However, there was an effect of group on *P*_max_ for both absolute and relative to body weight values and for *T*_opt_ (*d* = 1.2). Post hoc analyses showed that *P*_max_ and *T*_opt_ were lower for BFRrG compared to BFRG (*p* < 0.001; *d* = 0.9 and *p* = 0.002; *d* = 1.2 respectively) and compared to HG (*p* = 0.007; *d* = 1.3 and 0.009; *d* = 1.2 respectively). Relative *P*_max_ was lower for BFRrG compared to BFRG (*p* = 0.010; *d* = 0.6).

**Table 2:** Results of the force-velocity relationship test

	HG		BFRG		BFRrG	CG	Time Main effect	Group Main effect	***Interaction***
***P***_**max **_**(W)**									
Pre	885.7 ± 144.0	*a*	786.3 ± 114.3	*a*	702.3 ± 116.8	810.8 ± 103.5	*p = 0.29*	p < 0.001*	*p = 0.48*
Post	872.1 ± 149.4		820.7 ± 83.2		715.2 ± 125.1	822.5 ± 136.5			
***P***_**max**_**k (W**^**.**^**kg**^**−1**^**)**									
Pre	11.0 ± 1.3		10.2 ± 1.4	*a*	9.5 ± 1.3	10.4 ± 1.2	*p = 0.27*	p = 0.004*	*p = 0.55*
Post	10.9 ± 1.6		10.7 ± 1.2		9.7 ± 1.6	10.6 ± 1.7			
***T***_**0 **_**(N**^**.**^**m**^**−1**^**)**									
Pre	132.1 ± 32.6		124.6 ± 15.4		115.8 ± 20.0	121.2 ± 16.5	*p = 0.08*	*p = 0.19*	*p = 0.48*
Post	136.6 ± 26.1		141.2 ± 18.6		116.5 ± 22.5	129.0 ± 15.7			
***v***_**0 **_**(rpm)**									
Pre	231.1 ± 30.9		225.9 ± 18.5		228.0 ± 29.4	241.1 ± 15.9	*p=0.20*	*p = 0.34*	*p = 0.84*
Post	240.6 ± 30.0		223.3 ± 30.9		234.4 ± 32.3	251.1 ± 32.0			
***T***_**opt **_**(N**^**.**^**m**^**−1**^**)**									
Pre	76.2 ± 13.4	*a*	69.8 ± 8.0	*a*	60.4 ± 10.0	69.1 ± 7.1	*p = 0.97*	p < 0.001*	*p = 0.42*
Post	73.8 ± 14.2		74.4 ± 9.5		60.4 ± 12.2	72.6 ± 9.1			
***v***_**opt **_**(rpm)**									
Pre	108.4 ± 9.6		107.7 ± 11.0		111.3 ± 9.2	113.4 ± 9.7	*p = 0.27*	*p = 0.68*	*p = 0.84*
Post	113.7 ± 8.4		108.1 ± 13.6		114.4 ± 17.3	112.2 ± 9.7			

Data are shown as mean ± standard deviation. *HG* (*N* = 10) training in hypoxia, *BFRG* (*N* = 10) training with blood flow restriction during exercise, *BFRrG* (*N* = 10) training with blood flow restriction during recovery, *CG* (*N* = 9) control group with normal training, *Time main effect* difference between pre- and post-tests, *Group main effect* difference between exercise modalities, *P*_*max*_ maximal power output, *P*_*max*_*k* maximal power output relative to body weight, *T*_*0*_, maximal torque, *v*_*0*_ maximal velocity, *T*_*opt*_ optimal torque, *V*_*opt*_ optimal velocity

*Significant main effect

^a^Significantly different from BFRrG

### Anaerobic capacity

An increase of $\dot{V}{O}_2 peak$ expressed relative to body mass was found in BFRrG (*p* = 0.030; *d* = 0.7) but this effect was no longer observed for absolute $\dot{V}{O}_2 peak$ values. The former represents an increase of 7.5%. Concerning maximal accumulated oxygen deficit, no interaction and no main effect of group were observed. However, a time effect was found with higher values observed after training. $\dot{V} Epeak$ was higher in HG compared to BFRrG (*p* = 0.019; *d* = 1.4). RER was higher in BFRG (*p* = 0.002; *d* = 1.4) and in CG (*p* = 0.007; *d* = 1.3) compared to HG. However, no difference was found between the groups for the other variables (*p* > 0.05; Tables 3 and 4). A training effect was observed for all the other variables (*p* < 0.05), excepting peak $\dot{V}E$/$\dot{V}{O}_2$ and HRpeak.

**Table 3:** Results of the maximal anaerobic cycling test

	HG		BFRG		BFRrG		CG	Time Main effect	Group Main effect	***Interaction***
**Duration (s)**										
Pre	153.4 ± 25.5		132.2 ± 17.4		132.6 ± 27.4		131.0 ± 21.5	p < 0.001*	*p = 0.10*	*p = 0.95*
Post	177.3 ± 34.0		160.0 ± 35.8		154.8 ± 31.5		152.6 ± 44.1			
$\dot{\boldsymbol{V}}{\boldsymbol{O}}_{\mathbf{2}}\boldsymbol{peak}$** (mL**·**min**^**−1**^·**kg**^**−1**^**)**										
Pre	59.8 ± 7.5		51.9 ± 4.6		54.2 ± 5.8	*d*	54.0 ± 6.9	p = 0.004*	*p = 0.29*	p = 0.034*
Post	59.3 ± 6.5		54.8 ± 7.7		58.3 ± 6.5		54.9 ± 7.3			
$\dot{\boldsymbol{V}}{\boldsymbol{O}}_{\mathbf{2}}\boldsymbol{peak}$** (L**·**min**^**−1**^**)**										
Pre	4.62 ± 0.47		4.01 ± 0.44		3.99 ± 0.48		4.17 ± 0.49	p = 0.005*	*p = 0.06*	*p = 0.08*
Post	4.64 ± 0.42		4.22 ± 0.60		4.29 ± 0.54		4.24 ± 0.54			
**Accumulated O**_**2**_**uptake (mL**·**kg**^**−1**^**)**										
Pre	117.1 ± 35.9	*c*	83.5 ± 14.5		86.3 ± 27.2		85.7 ± 21.5	p < 0.001*	p = 0.038*	*p = 0.97*
Post	136.3 ± 28.6		108.5 ± 29.1		110.6 ± 34.5		104.6 ± 42.4			
**Accumulated O**_**2**_**uptake (L)**										
Pre	9.00 ± 2.52		6.48 ± 1.32	b	6.29 ± 1.82		6.60 ± 1.61	p < 0.001*	p = 0.001*	*p = 0.96*
Post	11.41 ± 3.48		8.39 ± 2.27		8.07 ± 2.41		8.02 ± 3.17			
**maxO**_**2**_**def (mL**·**kg**^**−1**^**)**										
Pre	43.7 ± 8.5		35.8 ± 7.4		39.6 ± 7.2		37.8 ± 7.3	p = 0.002*	*p = 0.44*	*p = 0.57*
Post	45.4 ± 7.5		42.6 ± 10.8		46.9 ± 6.2		42.4 ± 10.5			
**maxO**_**2**_**def (L)**										
Pre	3.41 ± 0.76		2.76 ± 0.57		2.92 ± 0.60		2.91 ± 0.51	p = 0.002*	*p = 0.22*	*p = 0.79*
Post	3.67 ± 0.81		3.27 ± 0.80		3.46 ± 0.55		3.26 ± 0.75			
**Aerobic (%)**										
Pre	72.2 ± 4.3	*c*	70.0 ± 3.5		67.2 ± 7.1		69.0 ± 4.6	p = 0.049*	p = 0.025*	*p = 0.88*
Post	74.7 ± 3.5		71.5 ± 5.1		69.0 ± 6.3		70.0 ± 5.5			
**Anaerobic (%)**										
Pre	27.8 ± 4.3	*c*	30.0 ± 3.5		32.8 ± 7.1		31.0 ± 4.6	p = 0.049*	p = 0.025*	*p = 0.88*
Post	25.3 ± 3.5		28.5 ± 5.1		31.0 ± 6.3		30.0 ± 5.5			
**%**$\dot{\boldsymbol{V}}{\boldsymbol{O}}_{\mathbf{2}}$**peak**										
Pre	98.7 ± 2.8		97.5 ± 6.9		95.8 ± 7.0		95.6 ± 6.0	p = 0.037*	*p = 0.73*	*p = 0.82*
Post	100.2 ± 2.8		99.1 ± 7.6		99.6 ± 7.6		98.9 ± 3.2			
**Mean **$\dot{\boldsymbol{V}}{\boldsymbol{O}}_{\mathbf{2}}$** (mL**·**min**^**−1**^·**kg**^**−1**^**)**										
Pre	46.2 ± 9.8		41.8 ± 3.4		43.5 ± 4.6		43.4 ± 6.5	p = 0.008*	*p = 0.42*	*p = 0.77*
Post	48.3 ± 6.3		44.1 ± 5.7		47.3 ± 5.3		44.5 ± 6.6			
**Mean **$\dot{\boldsymbol{V}}{\boldsymbol{O}}_{\mathbf{2}}$** (L**·**min**^**−1**^**)**										
Pre	0.61 ± 0.18		0.55 ± 0.09		0.60 ± 0.11		0.56 ± 0.10	p = 0.049*	*p = 0.59*	*p = 0.65*
Post	0.62 ± 0.15		0.58 ± 0.11		0.65 ± 0.12		0.58 ± 0.10			

Data are shown as mean ± standard deviation

*HG* (*N* = 10) training in hypoxia, *BFRG* (*N* = 10) training with blood flow restriction during exercise, *BFRrG* (*N* = 10) training with blood flow restriction during recovery, *CG* (*N* = 9), control group with normal training, *Time main effect* difference between pre- and post-tests, *Group main effect* difference between exercise modalities,$\dot{V}{O}_2 peak$peak oxygen consumption, *maxO*_*2*_*def* maximal accumulated oxygen deficit, *Aerobic (%)* contribution of aerobic processes, *Anaerobic (%)* contribution of anaerobic processes, %$\dot{V}{O}_2 peak$percentage of peak oxygen consumption from the ramp incremental exercise

*Significant main effect

^b^Significantly different from HG

^c^Trend (compared to BFRrG)

^d^Significantly different from Pre values within the same group

**Table 4:** Cardiorespiratory responses at the end of the anaerobic cycling test

	HG	BFRG	BFRrG		CG		Time Main effect	Group Main effect	***Interaction***
$\dot{\boldsymbol{V}}\boldsymbol{Epeak}$** (L**·**min**^**−1**^**)**									
Pre	183.1 ± 18.1	163.4 ± 23.7	146.7 ± 29.0	*b*	165.3 ± 26.2		p = 0.028*	p = 0.028*	*p = 0.22*
Post	182.9 ± 16.9	174.5 ± 21.5	159.7 ± 19.8		165.1 ± 19.1				
$\dot{\boldsymbol{V}}\boldsymbol{E}$**/**$\dot{\boldsymbol{V}}\mathbf{O}$_**2**_									
Pre	37.3 ± 2.0	38.7 ± 3.1	35.6 ± 4.8		38.0 ± 3.4		*p = 0.36*	*p = 0.20*	*p = 0.91*
Post	37.7 ± 3.1	39.1 ± 3.1	36.5 ± 3.6		37.9 ± 2.9				
$\dot{\boldsymbol{V}}\boldsymbol{E}$**/**$\dot{\boldsymbol{V}}\mathbf{CO}$_**2**_									
Pre	32.5 ± 4.5	30.7 ± 3.3	29.8 ± 3.9		30.6 ± 2.3		p < 0.001*	*p = 0.09*	*p = 0.85*
Post	34.5 ± 2.4	32.7 ± 3.2	31.4 ± 3.6		31.6 ± 1.9				
**RER**									
Pre	1.15 ± 0.09	1.27 ± 0.08	1.20 ± 0.09	*b*	1.24 ± 0.06	*b*	p < 0.001*	p = 0.002*	*p = 0.70*
Post	1.09 ± 0.09	1.20 ± 0.05	1.16 ± 0.05		1.20 ± 0.06				
**HRpeak (bpm)**									
Pre	181.6 ± 15.3	179.0 ± 8.1	180.2 ± 9.8		175.4 ± 18.0		*p = 0.72*	*p = 0.66*	*p = 0.98*
Post	180.7 ± 13.0	178.6 ± 9.7	180.6 ± 10.1		173.6 ± 16.6				

Data are shown as mean ± standard deviation

*HG* (*N* = 10) training in hypoxia, *BFRG* (*N* = 10) training with blood flow restriction during exercise, *BFRrG* (*N* = 10) training with blood flow restriction during recovery, *CG* (N = 9) control group with normal training, *Time main effect* difference between pre- and post-tests, *Group main effect* difference between exercise modalities, $\dot{V} Epeak$ peak minute ventilation, $\dot{V}{O}_2$ oxygen consumption, $\dot{V}{CO}_2$ carbon dioxide elimination, *RER* respiratory exchange ratio, *HRpeak* peak heart frequency

*Significant main effect

^b^Significantly different from HG

## Discussion

The aim of this study was to compare the effects of RST with BFR or systemic hypoxia on performance in endurance-trained subjects during a very short-time window. The main result of the present study is that there are overall no significant differences among the groups after 2 weeks of RST. However, the data show that a 2-week RST program with three training sessions *per* week may promote gains of peak aerobic power, as well as maximal accumulated oxygen deficit and accumulated oxygen uptake during a supramaximal constant-intensity test. Nevertheless, time-trial performance was not enhanced by the current protocols.

### Training data

In the present study, there was no difference on training data (*i.e.* mean and peak power, total work) within the protocol between groups and training sessions. However, a decrease of fatigue index was observed following the first session in all the groups, probably through habituation. The present protocol could be too short (six sessions spread over 2 weeks) to observe any evolution of power output and total work. Indeed, in a fairly similar protocol, Montero and Lundby previously observed that 4 weeks of RST with three sessions per week enhanced repeated sprint performance in endurance-trained subjects [51]. However, no additional effect of RSH over training in normoxia was observed in tests performed in both hypoxic and normoxic groups [51]. Other studies observed that RST protocols may enhance resistance to fatigue and thus may increase the number of sprints performed [52]. Nonetheless, no difference between RST and RSH on total work, mean power output, RPE and pain in the legs were observed in a protocol close to ours [7]. Finally, since significant interaction for maximal power during training was found, the commitment to 2 weeks of HIT training between the four groups is not guaranteed. However, post hoc analyses did not reveal differences between groups, suggesting that there was no significant difference.

### Ramp test

The present data indicate that peak aerobic power and exercise duration were improved by exercise interventions. These results are in line with previous literature that generated consistent data to confirm that high intensity and sprint exercises promote gains in cardiorespiratory fitness [53]. Nonetheless, the addition of hypoxic and BFR stresses to the current RST protocol did not induce further benefits on anaerobic performance and most physiological variables. However, previous literature has reported some enhancement of molecular actors involved in glycolysis with RSH [7, 52]. RSH with voluntary hypoventilation at low lung volume due to the hypercapnic effect also promotes significant effects on blood lactate concentration and stroke volume [18, 54]. Finally, a significant impact of training was observed concerning minute ventilation and RER suggesting that RST may induce changes in muscle metaboreflex and chemoreflex activation [55, 56]. Importantly, ventilation and RER appeared higher for HG compared to BFRrG and RER was lower for HG compared to BRFG and CG. These data suggest that an impact on the ventilatory response to exercise may exist and potentially affect the perception of effort which could play a role in fatigue development.

The absence of training effects on $\dot{V}{O}_2 peak$ from the ramp incremental exercise (both relative and absolute values) in all the groups is in line with the previous results of Kasai et al. [57] who found enhanced peak and mean power outputs after RSH (FiO_2_ = 14.5%) during repeated sprint tests without any effect on $\dot{V}{O}_2\mathit{\max}$. Similar results on $\dot{V}{O}_2\mathit{\max}$ were obtained by Wang et al. with a protocol consisting of 3 × 5 × 10 s with 20 s active recovery [58]. Of note, some studies observed a small improvement in $\dot{V}{O}_2\mathit{\max}$ with RSH, however, RSH appears more related to the enhancement of repeated-sprint ability in team-sport and endurance athletes [6, 8]. In the present study, increases in peak aerobic power were found following RST but without additional effect with both systemic hypoxia and BFR. However, it was previously found that sprint exercise training, with longer sprint durations may improve aerobic fitness [59].

### Torque-velocity test

All the force-velocity parameters (i.e., *P*_max_, *T*_0,_*T*_opt_, *v*_0_, and *v*_opt_) remained unaltered after training independently of the group. This suggests that the present RST protocol did not alter the mechanical properties of skeletal muscles leading to increases in maximal power output and muscle efficiency within contraction. Of note, significant differences were observed for longer protocol training duration [60]. Alternatively, it can be hypothesized that three RSH sessions per week could generate fatigue accumulation, thus blunting optimal adaptations.

### Time trial

Finally, time trial performance was not improved by the present training protocol. This result can be explained by the fact that, as previously shown by Zinner et al., the latter is largely determined by $\dot{V}{O}_2\mathit{\max}$ which was unaffected in our study [61]. Indeed, the authors found, with a mechanistic approach, that the principal adaptive mechanism to HIT is the improvement in aerobic energy production, and that the role of $\dot{V}{O}_2\mathit{\max}$ as a limiting factor for endurance performance becomes even more important after training [61]. However, data indicate that the addition of a systemic hypoxic stress or BFR applied during the exercise bouts does not further improve the training effects on both anaerobic and aerobic performance compared to the control group in such a short period. Consistent with this, RST with additional stress did not elicit larger gains in absolute $\dot{V}{O}_2 peak$ during the supramaximal constant-intensity exercises.

### Anaerobic performance

According to the data, the current protocol leads to increases of both maximal accumulated oxygen deficit and accumulated $\dot{V}{O}_2$ during the supramaximal constant-intensity exercises in a short time window, as well as contribution of aerobic process. These data suggest that, even if there were few effects on other performance data, training in such a short period should induce metabolic adaptations leading to enhancing some determinants of anaerobic performance. Of note, it cannot be ruled out that peak aerobic power from the time trial exercise should be improved through improvement of both maximal accumulated oxygen deficit and accumulated $\dot{V}{O}_2$. Importantly, it was also recently observed that repeated sprint performance can be improved after only a 2-week RSH protocol in elite soccer players, international rugby union players and cross-country skiers [19, 20, 22]. Thus, our data are in line with these studies suggesting that 2-week RSH protocols may enhance some parameters of anaerobic performance. Finally, other investigations also observed improved repeated sprint ability with longer duration protocols, such as the study of Pramkratok and coworkers [62] with a protocol consisting of three sets of 6-s × 10 sprints at 140% of velocity at peak oxygen uptake on a treadmill, 3 days per week for 6 weeks.

### Limitations

In this study, the four different groups allowed several comparisons, however, the number of groups made it difficult to complete a crossover study. In addition, even if no differences were observed in baseline data, individual trainability may represent a limitation. Therefore, these data must be interpreted regarding these limitations, especially concerning the observed tendencies. Another concern should be that we did not perform repeated sprint ability tests to examine the effectiveness of the present protocol on this field aptitude.

## Conclusions and perspectives

In conclusion, no global differences were observed among the groups after 2 weeks of RST. However, the present study indicated that RST can be useful to enhance peak aerobic power or maximal accumulated oxygen deficit and *VO*_2_ during a supramaximal constant-intensity exercise in a very short preparation window in endurance-trained participants as it was previously found by other studies concerning repeated sprint ability. Importantly, the addition of hypoxic stress did not add further benefits on performance in this population. The mechanisms that mediate changes in human performance still represent an important challenge for further research. Specifically, additional investigations are needed to compare the effects of different hypoxic protocols (e.g., systemic vs. local hypoxia) on field performance and to examine cellular adaptations occurring in response to these training modalities. Indeed, while systemic hypoxia only reduces the O_2_ availability to the working muscle, BFR may additionally affect training adaptations by trapping metabolites in the muscle. The mechanisms of action of systemic hypoxia and BFR may be different, making it important to encourage additional studies identifying generic and specific induced adaptations for a better exercise prescription.
